# Proceedings of the Medical Journal Association

**Published:** 1871-05

**Authors:** 


### Proceedings of the Medical Journal Association.

At 10 o'clock yesterday morning the Medical Journal Association met at the rooms of the San Francisco Medical Society on Sutter street. Dr. H. R. Storer, of Boston, President of the Association, called the meeting to order, and stated that from the arrangements being made the Delegates of the Medical Asssciation from the East would have a pleasant time.

In the absence of the Secretary, Dr. Henry Gibbons, Jr., was elected Secretary, *pro tem.*

The proceedings of the last annual meeting not being at hand,

Dr. N. S. Davis, of Chicago, read the plan of organization of the Society, the object of which is to cultivate fraternal relations among members of the medical profession, to urge a higher standard of preliminary education of persons proposing to enter the profession and to collate vital statistics.

On motion, the committee on Foreign Exchanges, consisting of Drs. Dawson, of New York, Parvin, of Louisville, and Mitchell of New Orleans, was continued, and Dr. Jones, of New Orleans, was added to the committee.

The President stated that he had communicated with Professor Henry, of the Smithsonian Institute, who had promised to aid in keeping up the International exchanges. The Chair also stated that last year the Society only numbered twelve or thirteen journals, but at present numbered about forty members.

RESOLUTIONS.

Dr. Davis offered the following resolutions:

*Resolved*, That the social, educational and scientific interests of the profession would be greatly promoted by a more complete organization in every State and district in our country, such organization being calculated not only to direct and diffuse knowledge, but also to afford the most efficient means for procuring concerted and efficient action on all important questions of medical education and progress.

*Resolved*, That deficiency in the general education of young men entering upon the study of medicine in this county is an event of great magnitude, not only constituting a barrier to individual progress in professional life, but greatly lessoning the general reputation and usefulness of the profession.

*Resolved*, That the members of this Association be requested to use their respective medical periodicals as agencies for calling the special attention of the profession to the topics mentioned in the foregoing resolutions, until such a professional sentiment is created that no regular practitioner will feel at liberty to receive a student into his office who does not present testimonials from some competent source that he has at least a competent knowledge of the ordinary branches of education, including the lower mathematics and the natural sciences ; and the several organizations are so far complete that the several State Societies become the real and authoritative representatives of the profession in each State.

On motion, they were laid over until the evening session.

EXTRACT FROM THE ADDRESS DELIVERED AT THE ANNUAL MEETING OF THE ASSOCIATION OF MEDICAL EDITORS AT SAN FRANCISCO, BY HORATIO ROBINSON STORER, M. D., PRESIDENT OF THE ASSOCIATION.

*Gentlemen of the Association of American Medical Editors:*

Coming together from the opposite portions of the Continent, we have met to-night, not merely "to cultivate professional courtesies and to facilitate the conduct and general management of our journals," but, still further to quote the language of our constitution, "to promote their usefulness, and make them a still greater power for professional and popular good, and thereby, most especially, to advance the interests of Medicine." Such being the purpose and intent of our organization, there can be no topic more appropriate for me to present to you, none more fitting to the time, the place, and all the circumstances of the occasion, than

THE MUTUAL RELATIONS OF THE MEDICAL PROFESSION, ITS PRESS AND THE COMMUNITY.

These relations are manifold. To consider them all would be impossible in the brief space of an half hour's address. I shall, therefore, endeavor to speak only of the most important of them, and, avoiding all attempt at fine writing to make my remarks terse, very plain, and thereby, I trust, effective.

The Medical Profession in this country consists of what? To

To this question a multiplicity of answers present themselves; all of them true to a certain extent, and yet all of them, save one, very degrading to the term's highest idea. Were every physician what he should be—a thoroughly honest, straightforward man, anxious only for his patients' welfare, laboring for the developement of his science and not alone for gain, liberalized by education, humanized in the highest sense by a constant entering into the sufferings he is compelled to meet, and, above and beyond all else, spiritualized by the recognition that his every success is but a vouchsafement of God's great mercy, and he but its humble instrument—what a different art were medicine, what a different place in the world.

Of the seventy thousand or more persons in the United States licensed under the Revenue laws to practice medicine, how large a proportion, is it supposed, can be claimed to possess the qualifications just adverted to? Even if we eliminate all who, in default of professional graduation, have no valid title to the name, and all professional empirics, of whatever stripe or hue—Caucasian, aboriginal, or Chinese; tackers, whether of "path" or "ist" to their names—there still remains a mighty host, swelled again to its original dimensions, if the title is permitted, as in many sections of the country, to dispensing druggists, and still again to that doubtful sex wearing the habiliments of womanhood, but assuming the work and the prerogatives, while it seeks to escape the legal responsibilities of man. * * * * * * *

That the education of physicians is frequently so limited goes far, there can be no doubt, to prevent that general bestowal of confidence which otherwise would be conferred. For this, however, the community partly, as in part ourselves, are to blame. If a sec nd-rate article is all that is sought by the purchaser, he should no complain if it be received. If the medical colleges are content to underbid each other, and year after year to pursue the suicidal warfare, they should not grieve that their students, become practitioners, so often are starvelings, and so frequently do them discredit.

Professional "intuition" in the treatment of disease is seldom to be found. It is a very different thing from the *vocation* of which I have already spoken—without a sense of which none should ever assume so sacred a trust. A knowledge of human nature is useful to us, as a matter of course. It no more, however, constitutes a complete preparation for practice thon would a knowledge of mechanics, or of inorganic analysis. It is as with houses built upon a rock and upon the sand,—unless early education be well laid and solid, a broad foundation, and the most elaborate after-structare will prove easily shaken and unsafe. It matters not what, or how many, the apparent exceptions to this rule, for these brilliant self-made men would have shone with far more lustre had they received the early training of whose lack none are more painfully conscious than themselves. However great is the credit their due, there's always a blur to the gem, and cometimes the very contrast with what might have been, makes this seem the greater. President Eliot, of Harvard University, told but the truth in that now famous paper of his, upon "The New Education." "The term "'learned profession,'" he said, "is getting to have a sarcastic flavor. Only a very small proportion of lawyers, doctors, and ministers, the country over, are Bachelors of Arts. The degrees of LL. B. and M. D. stand, on the average, for decidedly less culture than the degree of A. B., and it is found quite possible to prepare young men of scanty education to be successful pulpit exhorters in a year or eighteen months. A really learned minister is almost as rare as a logical sermon." And as for the yearly graduates from our medical schoools, "Poor humanity," continues President Eliot, "shudder at the spectacle of so large a crop of such doctors." Who of you will not admitt that a really learned physician, in the highest sense, is as rare as, by differentiation, the only possible method, a perfectly correct abdominal diagnosis,—which, I am sometimes inclined to say, has never yet been made.

Such being the truth, what of ourselves—to a certain extent representative members of the profession—and of the power which we yield, the press? As individuals we may be far from the standard our responsibilities demand—many of us undoubtedly are—but, in the aggregate, there's a mightiness in this editorial function, that makes one's chair well-nigh the throne of Jove. Woe to the evil-doers upon whom its bolts chance to descend!

This address is full of useful suggestion, and we regret our space will not allow us to present it entire to our readers.—Ed.

